# Exploring and Verifying the Mechanism and Targets of Shenqi Pill in the Treatment of Nonalcoholic Steatohepatitis via Network Pharmacology and Experiments

**DOI:** 10.1155/2022/6588144

**Published:** 2022-06-12

**Authors:** Beihui He, Zheng Chen, Yunmeng Nie, Minmin Luo, Sumei Xu, Junbin Yan, Zhiyun Chen

**Affiliations:** ^1^The First Affiliated Hospital of Zhejiang Chinese Medical University (Zhejiang Provincial Hospital of Traditional Chinese Medicine), Hangzhou, China; ^2^The Second Central Laboratory, Key Lab of Integrative Chinese and Western Medicine for the Diagnosis and Treatment of Circulatory Diseases of Zhejiang Province, The First Affiliated Hospital of Zhejiang Chinese Medical University, Hangzhou, China

## Abstract

Shenqi pill (SQP), a famous traditional Chinese medicine (TCM) herbal formula derived from Jinguiyaolue (Synopsis of Prescriptions of the Golden Chamber), has long been used to treat kidney yang deficiency syndrome. According to the TCM treatment principle that the liver and kidney are homologies, the clinical use of SQP in the treatment of nonalcoholic steatohepatitis (NASH) has achieved a good effect. However, the active targeted genes and underlying mechanism remain unclear. In this study, we aimed to explore the treatment mechanism of SQP in NASH rats, which may further contribute to the in-depth exploration of SQP in clinical applications. Network pharmacology analysis was used to screen the target genes of SQP for NASH treatment based on public databases. Gene Ontology (GO), Kyoto Encyclopedia of Genes and Genomes (KEGG) enrichment analysis, and protein–protein interaction (PPI) analysis were used to search for crucial target genes and mechanisms. UPLC–MS/MS was used to verify the active compounds of the SQP screened. The hepatic pathology and biochemical indicators of rats were used to judge the modeling results and the curative effect of SQP. Western blotting and qRT–PCR were used to verify the expression of crucial target genes at the protein and RNA levels, respectively. Network pharmacology analysis and bioinformatics analysis showed that PTGS2, JUN, MYC, and CDKN1A might be crucial target genes in the primary mechanism of SQP in treating NASH and improving the inflammatory response. The UPLC–MS/MS results confirmed that the hub active compound, quercetin, screened out through the TCMSP database, is indeed present in SQP. Hepatic injury and lipid metabolism indicators of NASH rats were significantly improved after SQP treatment. The results of WB and qRT–PCR showed that the expression of PTGS2, JUN, MYC, and CDKN1A was higher in NASH rats than in normal rats and decreased after SQP treatment. The expression of inflammatory cytokines (IL-1*β*, IL-6, TNF-*α*) was reduced after SQP treatment, which confirmed that SQP could improve hepatic inflammation in rats. These results suggested that SQP could ameliorate NASH in rats, and that quercetin may be the critical active compound that exerts the therapeutic effect.

## 1. Introduction

Nonalcoholic fatty liver disease (NAFLD), a liver injury disease caused by metabolic disorders [1], includes nonalcoholic fatty liver (NAFL), nonalcoholic steatohepatitis (NASH), liver fibrosis and cirrhosis, and even hepatocellular carcinoma (HCC) [2]. NASH is associated with metabolic syndrome and diabetes mellitus (DM), which promotes the onset of arteriosclerotic vascular disease and a variety of extrahepatic malignant carcinomas [3]. Coupled with its high incidence and prevalence, NASH is causing a significant economic and health burden globally [4, 5].

NASH patients could be improved by control measures such as exercise and diet adjustment. However, it is difficult to adhere to these measures. Additionally, compared with NAFL, NASH accompanied by inflammation is more challenging to reverse [6]. It is particularly crucial to clarify the pathogenic mechanism of NASH and to seek effective drugs for its treatment.

In the cognition of traditional Chinese medicine (TCM), according to its clinical manifestations, NASH belongs to the categories of “Xie-tong” (hypochondriac pain) and “Ji-ju” (accumulation). The statistical results showed that the median age of the NASH population is 55 years old, which means that NASH patients are mostly middle-aged and elderly [7]. Chinese medicine believes that spleen and kidney functions will gradually decline in elderly individuals. Weak spleen and kidney functions will lead to disordered Qi movement, stagnation of water, dampness, phlegm, and stasis, which eventually hurt the liver. Therefore, tonifying the kidney and strengthening the spleen is one of the principles of the clinical treatment of NASH in TCM. Shenqi pill (SQP), a representative formula for spleen/kidney invigoration, comes from the “Jinguiyaolue (Synopsis of Prescriptions of the Golden Chamber),” written by Zhongjing Zhang, a Chinese medicine expert in the Han dynasty. The spleen/kidney tonifying function of SQP has been verified by various experiments [8, 9]. According to the TCM treatment principle that the liver and kidney are homologies, the clinical use of SQP in the treatment of NASH has achieved good effects [10, 11].

SQP is comprised of Cinnamomi ramulus (*Guizhi*), Aconiti Lateralis Radix Praeparata (*Fuzi*), Rehmanniae Radix Praeparata (*Dihuang*), Cornus officinalis Sieb. et Zucc. (*Shanzhuyu*), Cortex Moutan (*Mudanpi*), Rhizoma Dioscoreae (*Shanyao*), Poria cocos (*Fuling*), and Alisma orientale (*Zexie*). The herbal name has been checked by The Plant List (http://www.theplantlist.org) and Chinese Pharmacopoeia (http://db.ouryao.com/yd2015/).

**Figure 1:**
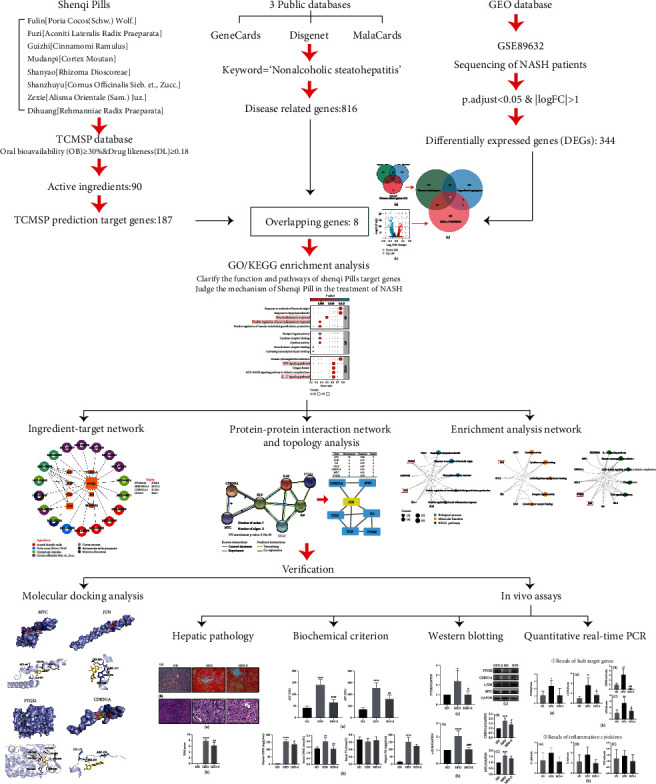
The analysis flow of the study.

At present, many studies have confirmed that SQP has an excellent curative effect in the treatment of diseases related to spleen and kidney yang deficiency [12–14]. This research group's long-term clinical practice also found that SQP has excellent efficacy in the treatment of NASH patients because of the effects of SQP in invigorating the spleen/kidney. A previous study by our team also suggested that SQP has superior efficacy in the treatment of NASH in rats [15]. SQP is comprised of multiple herbs that have the advantage of numerous curative effects, which also makes it challenging to clarify the mechanism of SQP in the treatment of NASH. Therefore, in this study, we used network pharmacology, molecular docking analysis, and in vivo experimental verification in rats to explore the mechanism and targets of SQP in the treatment of NASH, which may provide theoretical support for the clinical promotion of SQP. The analysis process of the study is shown in Figure 1.

## 2. Materials and Methods

### 2.1. Screening the Crucial Treatment Targets/Mechanism of SQP

#### 2.1.1. Screening of Active Compounds in SQP

The active compounds of SQP were queried through the Traditional Chinese Medicine Database and Analysis Platform (TCMSP) (https://tcmsp-e.com) in combination with oral bioavailability (OB) and drug-like (DL) [16]. The screening conditions were OB ≥ 30% and DL ≥ 0.18. OB refers to the speed and extent of oral drugs absorbed from the gastrointestinal tract and reaching circulation. The OB value of the compounds was predicted based on the OBioavail 1.1 analysis model [17]. DL refers to the similarity of a compound to a known drug. In this study, the DL index of new compounds was calculated according to the Tanimoto coefficient [18]. The calculation formula is as follows:

(1) $$\begin{array}{c} ƒ\left(A,B\right)=\frac{A*B}{{\left|A\right|}^{2}+{\left|B\right|}^{2}-A*B}. \end{array}$$

*A* represents the measured compounds. *B* is a fixed value representing the mean DL index of all known components of the Drugbank database (https://drugbank.ca).

A combination of these indicators can effectively select the bioactive compounds of SQP.

#### 2.1.2. Screening of Targets of SQP in the Treatment of NASH

(i) Screening the predicted target genes of active compounds through the TCMSP database is as follows; **(**ii) GSE89632, a NASH-related sequencing dataset, was downloaded from the Gene Expression Omnibus (GEO) database to screen the differentially expressed genes (DEGs) between NASH patients and healthy adults; and (iii) “Nonalcoholic steatohepatitis” was used as a keyword to screen NASH-related disease genes in the DisGeNET (https://www.disgenet.org/home), GeneCards (https://www.genecards.org), and MalaCards (https://www.malacards.org/pages/info) databases. The intersection of the above results was regarded as the target genes of SQP in treating NASH

#### 2.1.3. Clarifying the Mechanism of SQP in the Treatment of NASH

The *R* package cluster Profiler [19] was used to perform Gene Ontology (GO) and Kyoto Encyclopedia of Genes and Genomes (KEGG) enrichment analyses on the screened target genes of SQP to treat NASH. Gene Ontology contains three ontologies, which describe the molecular function (MF), cellular component (CC), and biological process (BP) of the gene. It is an internationally standardized functional classification system to comprehensively describe the attributes of genes. KEGG enrichment analysis was used to clarify SQP target gene-related pathways based on the KEGG database.

Combined with the gene functions and pathways of SQP target genes, we can explain the primary mechanism of the above genes and then clarify the mechanism of SQP in treating NASH, which is also helpful for the subsequent screening of hub genes.

### 2.2. Screening Hub Genes of SQP in Treating NASH

#### 2.2.1. Construction of the “Compound-Target” Network

To clarify the relationship between the active compounds and targets of SQP, the screened compounds and therapeutic targets of SQP were connected into a “compounds-target” network.

We could determine which active compounds in SQP play a significant treatment role and clarify which hub target genes are affected by multiple active compounds simultaneously through the network.

#### 2.2.2. Construction of the “Protein–Protein Interaction” Network and Topology Analysis

The STRING (https://string-db.org) database was used to clarify the protein interaction relationship of SQP target genes (the screening threshold was interaction score confidence ≥ 0.9). CytoNCA, the plug-in of Cytoscape 3.8.0, was used for network topology analysis according to degree centrality (DC), betweenness centrality (BC), and closeness centrality (CC) to screen the hub target genes at the core of the “protein–protein interaction (PPI)” network.

#### 2.2.3. Construction of “Enrichment Analysis” Network

An “enrichment analysis” network was constructed to display the specific gene functions and pathways involved in the treatment of target genes through the enrichment analysis results and related genes. Therefore, we could determine whether the selected hub target genes are associated with the mechanism of SQP in treating NASH.

### 2.3. UPLC–MS/MS

Ultraperformance liquid chromatography-tandem mass spectrometry (UPLC–MS/MS) was used to verify the previously screened crucial compounds of SQP existing in various Chinese herbs or acting on multiple target genes. This verification process could clarify the exact compounds of SQP and further judge the reliability of the results selected based on the public databases.

UPLC–MS/MS was performed at the Academy of Chinese Medicine, Zhejiang Chinese Medical University. The SQP extracts were dissolved in 80% methanol to a concentration of 10 mg/ml, filtered through a 0.22 *μ*m membrane, and separated on a CORTECS UPLC T3 column (2.1 × 100 mm, 1.6 *μ*m) at 4°C. The mobile phases consisted of solvents A (0.1% formic acid) and B (acetonitrile). Gradient elution is as follows: 0–2.00 (min), A: 5%; B: 95%; 2–32.00 (min), A: 100%; 32–33.00 (min), A: 100%; 33–33.50 (min), A: 5%; B: 95%; 33.5–35.00 (min), A: 5%; and B: 95%. The total running time was 35 min. The solvent flow rate was 0.3 ml/min. The column temperature was 30°C, and the injection volume was 2 *μ*l.

The MS monitoring was run in both positive ion mode and negative ion mode. For positive ion mode, the heater temperature was 325°C, the sheath gas flow rate was 45 arb, the aux gas flow rate was 15 arb, the sweep gas flow rate was 1 arb, the spray voltage was 3.5 kV, the capillary temperature was 330°C, and the S-Lens RF level was 55%. For negative ion mode, the heater temperature was 325°C, the sheath gas flow rate was 45 arb, the aux gas flow rate was 15 arb, the sweep gas flow rate was 1 arb, the spray voltage was 3.5 kV, the capillary temperature was 330°C, and the S-Lens RF level was 55%. The MS scan mode was Full Scan (m/z 100–1,500). The MS/MS scan mode was set as dd-MS2 (TopN = 10). MS spectra were acquired at a resolution of 120,000, and MS/MS scans were acquired at 60,000.

Finally, we compared the mass charge ratio (m/z) and structure of the active compound to determine whether the above components, which were selected via the TCMSP database, were actually present in SQP. A deviation range between 5 m Dalton is accepted. The molecular weight and structural formula of the compounds were referenced in the PubChem database, which records chemical information from authoritative sources (https://pubchem.ncbi.nlm.nih.gov/).

### 2.4. Molecular Docking Verification

AutoDock Vina 1.2.0 was used for molecular docking. The 3D structures of the crucial active compounds (ligands) of SQP were downloaded through PubChem (https://pubchem.ncbi.nlm.nih.gov), a database of small organic molecules' biological activity. We also downloaded the protein structure of the hub target genes (receptors) of SQP in the treatment of NASH through the Protein Data Bank (PDB) (http://www.rcsb.org), a protein structure database, and set up the active pocket of the receptor for subsequent molecular docking. A binding energy < 0 indicates that the ligand could bind with the receptor. The smaller the value of the binding energy is, the stronger the binding ability of the ligand to the receptor.

Then, the hydrogen bond interaction was analyzed. The atom covalently bonded to the hydrogen atom is the hydrogen donor, and the other electronegative atom is the hydrogen acceptor. By visualizing the results of molecular docking, the hydrogen acceptor/donor can be judged (the one with a lone pair of electrons is the acceptor, with a couple of electrons being the donor). Subsequently, we referred to the Lipinski (rule of five) principle [20] (small molecule drugs must have the following properties: (I) the molecular weight is less than 500, (II) the number of hydrogen bond donors is less than 5, (III) the number of hydrogen bond acceptors is less than 10, (IV) the lipid-water partition coefficient is less than 5, and (V) the number of rotation keys does not exceed 10) to predict whether the crucial compounds of SQP can be further developed as small molecule drugs for hub target genes.

### 2.5. In Vivo Experimental Verification

#### 2.5.1. Formula Preparation

The Traditional Chinese Medicine Preparation Room of The First Affiliated Hospital of Zhejiang Chinese Medical University made a liquid extract with an herbal ratio of 8 : 4 : 4 : 3 : 3 : 3:1 : 1. The specific dosages of SQP are Rehmanniae Radix Praeparata (*Dihuang*) 24 g, Rhizoma Dioscoreae (*Shanyao*) 12 g, Cornus officinalis Sieb. et Zucc. (*Shanzhuyu*) 12 g, Alisma orientale (*Zexie*) 9 g, Poria cocos (*Fuling*) 9 g, Cortex Moutan (*Mudanpi*) 9 g, Cinnamomi ramulus (*Guizhi*) 3 g, and Aconiti Lateralis Radix Praeparata (*Fuzi*) 3 g, as recorded in Jinguiyaolue (Synopsis of Prescriptions the Golden Chamber).

#### 2.5.2. Animal Modeling and Treatment

Male Sprague–Dawley (SD) rats (180 ± 10 grams, SPF) were purchased from Shanghai Sippe-Bk Lab Animal Co., Ltd. and were housed in the Experimental Animal Research Center of Zhejiang Chinese Medical University. The animal license was SCXK (Hu) 2008-0016. This study was approved by the Laboratory Animal Management and Ethics Committee of Zhejiang Chinese Medicine University (Resolution number: ZSLL-2010-26) and followed the guidelines of the National Guidelines for Experimental Animal Welfare.

After one week of adaptive feeding, 15 SD rats were randomly divided into three groups: the normal diet (ND) group, the high-fat diet (HFD) group, and the group treated with SQP (HFD-S), with five rats per group. ND group rats were fed a normal diet. The rats in the other groups were provided a high-fat diet of 82.5% normal diet, 10% lard, 2% cholesterol (Shanghai Bio Science & Technology Co., Ltd., lot: 130208), 0.5% sodium cholate (Hangzhou Microbial Reagent Co., Ltd., lot: 20130125-00), and 5% egg yolk powder. Meanwhile, rats in the HDF-S group were given 14 g·kg^−1^·d^−1^ SQP liquid extract for 12 weeks. The reason for the selected SQP dosage is to achieve a total of 81 g per SQP serving. Divide the total per serving of SQP by the weight of a typical adult (70 kg) to obtain an adult human dose of 1.15 g·kg^−1^. The rats were treated with intragastric administration at 12 times the normal human adult dose. Finally, 14 g·kg^−1^·d^−1^was obtained as the dose for rats in the study. The remaining two groups of rats were given an equal volume of normal saline for 12 weeks. After modeling, all rats were sacrificed under anesthesia. Serum and livers were collected and stored at -80°C until further use.

#### 2.5.3. Hepatic Pathology and Serum Biochemical Indicators

Serum alanine aminotransferase (ALT), aspartate aminotransferase (AST), triglyceride (TG), and total cholesterol (CHOL) were detected by an automatic biochemical analyzer. TG and CHOL levels in the rat liver were also detected. Hepatic pathology of the rat livers (steatosis/inflammation) was detected by Oil Red O and hematoxylin-eosin (HE) staining. The pathological diagnosis of NAFLD was based on the “Guidelines for the Diagnosis and Treatment of Nonalcoholic Fatty Liver Disease (2010 Revision),” and the NAFLD Activity Score (NAS) was calculated [21].

#### 2.5.4. Western Blotting

Rat hepatic proteins were extracted according to the instructions of the total protein extraction kit. The protein concentration was determined by a BCA kit and diluted to 5 *μ*g/*μ*l. Proteins (10 *μ*l) were loaded on 10% SDS–PAGE gels and transferred to PVDF membranes by the semidry transfer method. Then, the PVDF membranes were incubated overnight with the corresponding primary antibodies directed against CDKN1A (1 : 800), MYC (1 : 1000), JUN (1 : 600), and PTGS2 (1 : 2000) at 4°C, followed by incubation with a secondary antibody. An ECL Kit was used to reveal the protein binding. An Odyssey infrared imaging system was used to collect images and analyze the values.

The total protein extraction kit (cat: P1250; lot: 2021SOAP1250) was purchased from APPLYGEN. The BCA protein quantification kit (cat: PQ0012; lot: A00445) was purchased from MULTI SCIENCES. 2× SDS protein loading buffer (cat: P1018; lot: 20201027) was purchased from Solarbio. The Western ECL substrate kit (cat: 170-5060; lot: 102031512) was purchased from Bio-Rad. The prestaining Western Blot Marker (cat: C0302A; lot:201076) was purchased from Haigene. The anti-c-JUN antibody (cat: 9165S; lot:9) was purchased from Cell Signaling Technology. The anti-MYC (cat: 16286-1-AP; lot:00092831), anti-PTGS2 (cat: 66351-1-lg; lot: 10021385), and anti-GAPDH antibodies (cat: 60004-1-Ig; lot:10013030) were purchased from Proteintech. CDKN1A (cat: ER1906-07; lot: BN1203) was purchased from HUABIO. HRP-conjugated goat anti-rabbit IgG (H + L) (cat: GAR007; lot: A00613) and HRP-conjugated goat anti-mouse IgG (H + L) (cat: GAM007; lot: A160741) were purchased from Multi Science.

#### 2.5.5. Quantitative Real-Time PCR

Then, 400 *μ*l lysis buffer, three grinding beads, and 50 mg hepatic tissue were mixed in a grinding tube and subsequently broken with a homogenizer (5000 rpm, 20 seconds). The tube was then centrifuged (12000 rpm, 5 min, 4°C), and 300 *μ*l of supernatant was placed in a new 1.5 ml RNase-free tube. Next, RNA was extracted using a Universal RNA Extraction Kit. The reverse transcriptase reaction was performed for 15 min at 37°C and 5 seconds at 85°C. Finally, a 10 *μ*l PCR mix was applied, including 2 *μ*l cDNA, 5 *μ*l 2× TB green, 0.4 *μ*l forward primer (10 *μ*m), 0.4 *μ*l reverse primer (10 *μ*m), and 2.2 *μ*l ddH_2_O.

The Universal RNA Extraction kit (cat: 9767; lot: AJ82351A), reverse transcription (RT) kit (cat: RRO36A; lot: A140832A), and RNA amplification kit (cat: RR820A; lot: A140778A) were purchased from Takara.

We designed primer sequences according to the mRNA sequences obtained from the NCBI database (https://www.ncbi.nlm.nih.gov) and PrimerBank (https://pga.mgh.harvard.edu/primerbank/), synthesized by Sangon Biotech, as shown in Table 1.

### 2.6. Statistical Analysis

SPSS 21.0 and GraphPad Prism version 8 were used for data analysis and visualization. Data are expressed as the mean ± standard error (SE). One-way analysis of variance was used to analyze the statistical significance between the ND, HFD, and HFD-S groups. A *p* value <0.05 indicates that the result is statistically significant.

## 3. Results

### 3.1. Results of the Pharmacology Network Analysis

#### 3.1.1. The Compounds of SQP

From the TCMSP database, we screened 91 unique SQP active compounds of 8 Chinese herbs, meeting the screening conditions with OB ≥ 30% and DL ≥ 0.18 (Supplementary Table S1).

#### 3.1.2. The Target Genes of SQP in Treating NASH

(i) We searched the TCMSP database and found 182 predicted target genes of the SQP active compounds. (ii) Downloading the GSE89632 dataset from the GEO database, we selected NASH and HC (healthy control) samples and used *p*.adj < 0.05 and |logFC| > 1 as the threshold for screening DEGs. A total of 344 DEGs were screened. (iii) We used “non nonalcoholic steatohepatitis” as the keyword to screen NASH-related genes in the DisGeNET, GeneCards, and MalaCards databases. A total of 816 intersecting genes were called NASH-related disease genes.

**Figure 2:**
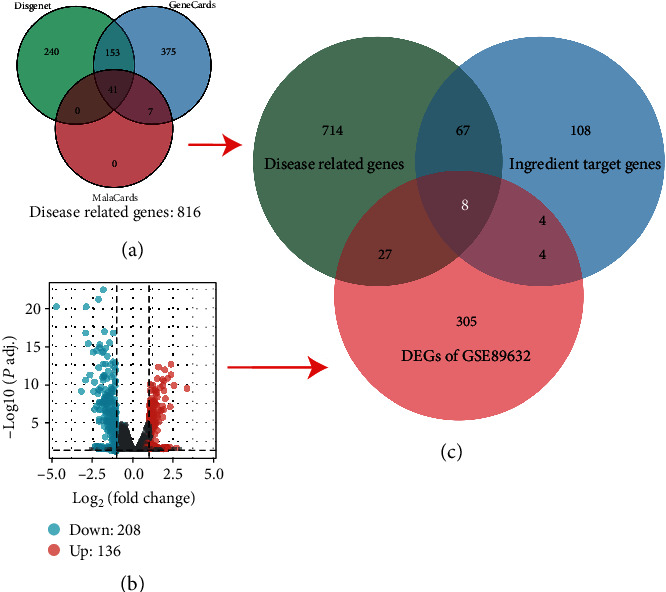
The process and results of the SQP target gene screening. (a) Screening results of NASH-related genes. (b) The screening results of DEGs. (c) The final screening results of the SQP target genes.

Then, we took the intersection of 187 predicted target genes of SQP compounds, 344 DEGs, and 816 NASH-related disease genes, which were seen as the target genes of SQP for the treatment of NASH (Figure 2). A total of 8 genes were selected as the target genes of SQP: prostaglandin-endoperoxide synthase 2 (PTGS2/COX-2), Jun oncogene (JUN/c-JUN), cyclin-dependent kinase inhibitor 1A (CDKN1A/P21), interleukin 6 (IL6), MYC protooncogene (MYC), interleukin 1 beta (IL1B), C-C motif chemokine ligand 2 (CCL2), and serpin family E member 1 (SERPINE1).

#### 3.1.3. The Mechanism of SQP in Treating NASH

**Figure 3:**
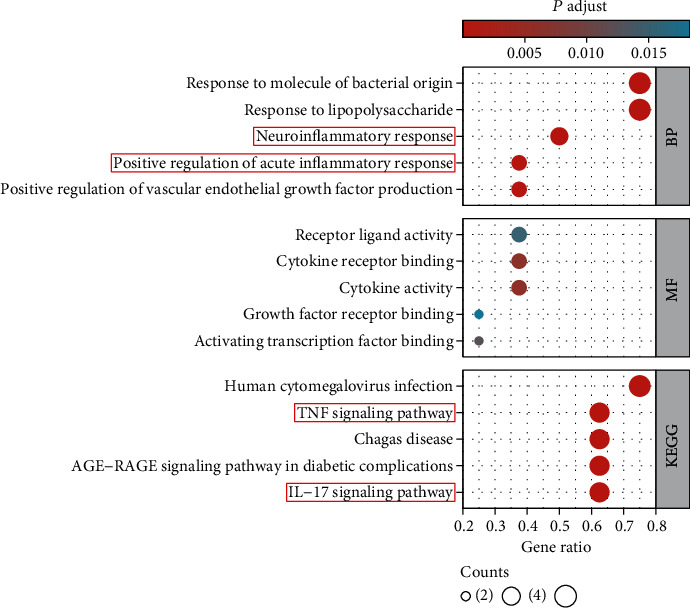
Enrichment analysis results (top 5).

To confirm the treatment mechanism of SQP, we performed GO and KEGG enrichment analyses on the above eight target genes. The *R* package clusterProfiler was used for enrichment analysis and visualization. The screening thresholds were *p*.adj < 0.05 and *q* value <0.05. The results showed that a total of 870 BP, 17 MF, 0 CC, and 70 pathways were identified. The top 5 enrichment results of each category are shown in Table 2. The ontologies of BP are related to response to lipopolysaccharide, neuroinflammatory response, and positive regulation of acute inflammatory response. The above BPs are both related to inflammation. Regarding MFs, cytokine activity and cytokine receptor binding had the lowest *p* value, indicating the highest credibility, and they may be associated with the secretion of inflammatory cytokines. The results of the KEGG enrichment analysis showed that the IL-17 signaling pathway and TNF signaling pathway with low *p* values were inflammation-related pathways (Figure 3). The enrichment analysis results of the SQP target genes suggest that the therapeutic mechanism of SQP's treatment of NASH may be to improve liver inflammation.

### 3.2. Hub Genes of SQP in Treating NASH

#### 3.2.1. Hub Genes That Interact the Most with SQP Compounds

**Figure 4:**
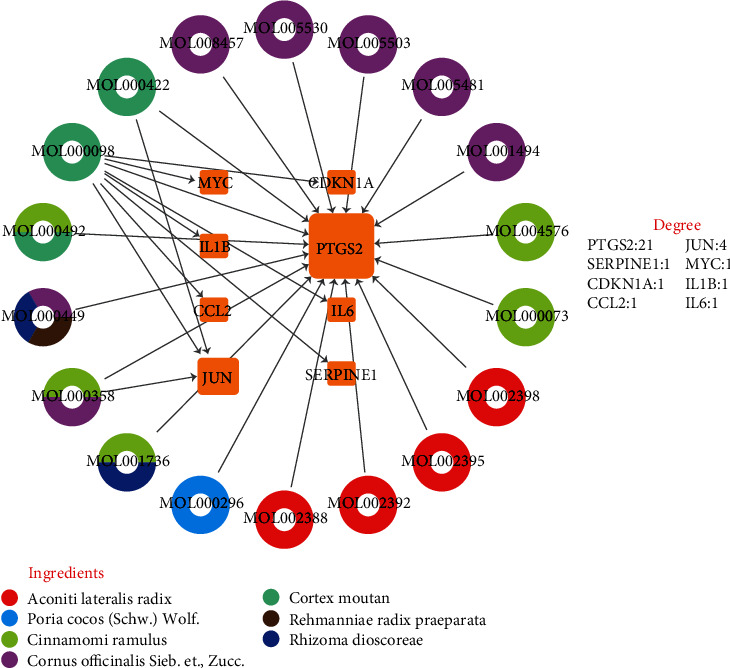
“Compound-target” network. The middle yellow squares represent the target genes of SQP; the larger the shape of the gene is, the more active compounds are targeting that gene. The outer circles represent active compounds, and different colors indicate that the active compound exists in the different Chinese herbs.

The active compounds and therapeutic targets of SQP were input into Cytoscape 3.8.0 software to construct the “compound-target” network (Figure 4). The colors indicate that the compound is present in multiple herbs simultaneously. The connection line demonstrates the relationship between the compounds and the target genes. The results support that NASH treatment by SQP is not based on a single component or targeting a single gene but by a combination of multiple compounds and genes, which is also the unique advantage of traditional Chinese medicine. However, not every herb will act on therapeutic target genes. Alisma orientale (Sam.) Juz. (Zexie) has no compounds that regulate the SQP target genes.

MOL000098 (quercetin) is the most crucial compound with the most targets. Quercetin could act on all eight selected target genes of SQP simultaneously. PTGS2 is the top gene affected by the most active compounds, suggesting that PTGS2 may be the hub gene of SQP in treating NASH.

#### 3.2.2. Genes at the Core of the PPI Network

**Figure 5:**
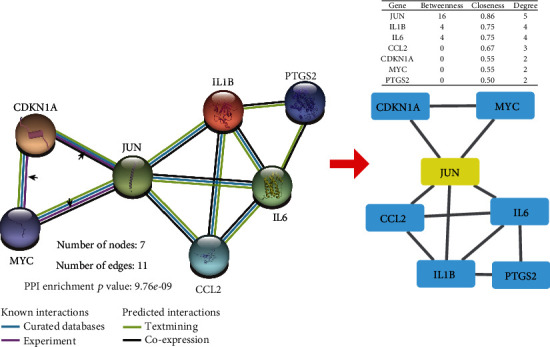
“Protein–protein interaction” network. JUN is the hub gene of this protein interaction network, and it has an exact protein interaction relationship with CDKN1A and MYC.

The above 8 target genes were input into the STRING database, and CytoNCA was used to calculate the gene connections and screen the core therapeutic genes according to DC, BC, and CC. The results showed that JUN was the hub gene with the highest DC, BC, and CC among the eight therapeutic target genes (Figure 5).

In addition, JUN has an exact protein interaction relationship with CDKN1A and MYC (determined by experiments [22–25]), suggesting that JUN may not only be the direct hub target gene of SQP but also further enhance the effect of SQP in improving inflammation by interacting with CDKN1A and MYC.

#### 3.2.3. Genes Related to Inflammation

**Figure 6:**
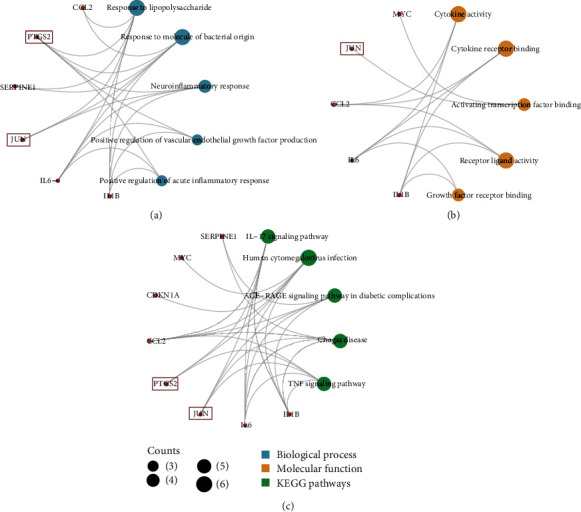
“Enrichment analysis” network. (a) The subnetwork of biological processes. (b) The subnetwork of molecular functions. (c) The subnetwork of KEGG pathways. The red nodes represent the targets gene of SQP, the blue nodes represent biological processes associated with these genes, the yellow nodes represent molecular functions associated with these genes, and the green nodes represent the KEGG pathways associated with these genes.

To further clarify whether the selected hub genes PTGS2 and JUN are related to the active mechanism of SQP in treating NASH by improving inflammation, we constructed an enrichment analysis network, showing that PTGS2 and JUN are both genes related to inflammation (Figure 6). CDKN1A and MYC, genes that interact with JUN, are also associated with inflammation.

Finally, we selected two hub target genes (PTGS2 and JUN) and two genes (MYC and CDKN1A) that may synergize with JUN to aggravate hepatic inflammation via the eight SQP target genes.

#### 3.2.4. Proposing the Hypothesis of the Therapeutic Mechanism of SQP

In summary, we speculated that PTGS2 (the crucial target of the active compounds of SQP) and JUN (the essential gene among the eight target genes of SQP) are the hub genes for the treatment of NASH. SQP may improve inflammation in the liver and reduce the secretion of inflammatory factors (IL1B, IL6, TNF-*α*) by regulating the expression of PTGS2 and JUN and affecting the interactions of JUN, CDKN1A, and MYC.

### 3.3. Results of UPLC–MS/MS

**Figure 7:**
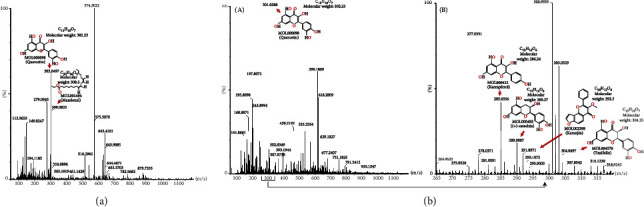
The results of UPLC–MS/MS. (a) The MS monitoring was run in positive ion mode. (b), (A) The MS monitoring was run in negative ion mode. (B) MS results for compounds with m/z between 265 and 320.

UPLC–MS/MS was used to determine whether the above crucial compound, shown in the “Compound-target” network, existed in SQP to support the reliability of the screening results from the public databases. In negative ion mode, we found that there may be some potential target compounds in the 265-320 mass charge ratio range; so, we further analyzed the compounds in the range of 265-320 m/s.  Figure 7 shows that the active compounds of SQP predicted by the TCMSP databases, such as MOL001494 (mandenol), MOL000098 (quercetin), MOL000422 (kaempferol), MOL000492 ((+)-catechin), MOL002398 (karanjin), and MOL004576 (taxifolin), were found in UPLC–MS/MS analysis results, which also shows that SQP is indeed acting via multiple compounds simultaneously to treat the NASH rats. However, not all predicted active compounds were selected in the results. The analysis results suggest that the prediction results of the active compounds based on the TCMSP database have a certain accuracy but are not entirely accurate. Subsequent UPLC–MS/MS analysis is necessary.

### 3.4. Results of Molecular Docking Analysis

We found that quercetin is the crucial compound of SQP because it acts on multiple target genes (PTGS2, JUN, CDKN1A, IL6, MYC, IL1B, CCL2, and SERPINE1). Therefore, we selected quercetin and four crucial SQP target genes for molecular docking verification. As shown in Table 3, the values of the minimum binding energy of quercetin and the hub target genes of SQP were all less than 0, indicating that quercetin, the crucial compound of SQP, could combine with PTGS2, JUN, MYC, and CDKN1A, achieving curative effects. Among them, PTGS2 and quercetin showed a robust potential affinity with a binding energy of -9.1 kcal/mol, while the remaining three target genes showed a significantly weaker affinity with quercetin than PTGS2. The above results also further support the “compound-target” network, indicating PTGS2 is a crucial target gene for SQP with the most interactions with active ingredients.

**Figure 8:**
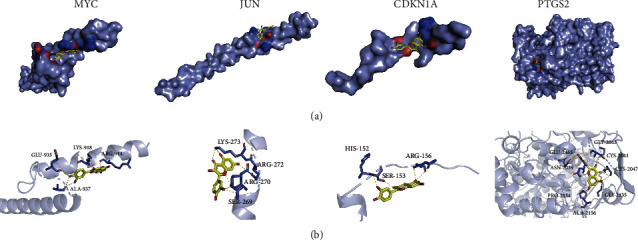
Molecular docking results of quercetin and the hub target genes. (a) The 3D structure of the molecular docking. (b) The hydrogen bond interactions of the molecular docking.

Furthermore, we analyzed the docking results of quercetin and the hub target genes.  Figure 8(a) shows the 3D visualization of the docking results. It can be seen from the figure that quercetin is embedded in the hollow recesses of the MYC (6G6J), JUN (1JNM), CDKN1A (4RJF), and PTGS2 (1CVU) protein structures, which is similar to the theoretical active pocket morphology; so, the docking result can be preliminarily believed to be theoretically reliable. Subsequently, the hydrogen bond interaction was analyzed (Figure 8(b)). MYC formed hydrogen bond interactions with quercetin through its GLU-935, LYS-918, ARG-914, and ALA-937; JUN interacted with quercetin through LYS-273, ARG-272, ARG-270, and SER-269; CDKN1A combined with quercetin via HIS-152, ARG-156, and SER-153; and PTGS2 formed hydrogen bond interactions with quercetin through GLU-2465, GLY-2045, ASN-2039, CYS-2041, CYS-2047, PRO-2154, ALA-2156, and GLY-2135.

The hydrogen acceptor/donor can be calculated according to the molecular docking visualization (Figure 8(b)). The results show that (i) quercetin and CDKN1A (4RJF) have two hydrogen donors and two hydrogen acceptors, (ii) the combination of quercetin and JUN (1JNM) has three hydrogen donors and two hydrogen acceptors, (iii) quercetin and MYC (6G6J) have nine hydrogen donors and three hydrogen acceptors, and (iv) quercetin and PTGS2 (1CVU) have ten hydrogen donors and seven hydrogen acceptors. Combining the number of hydrogen bond donors and acceptors, referring to the Lipinski (rule of five) principle of drugs, we found that, in theory, quercetin is suitable for development as a small molecule drug targeting JUN, CDKN1A, and MYC but it is not suitable for targeting PTGS2. Although quercetin cannot be developed as a small molecule drug that acts independently of PTGS2, its efficacy is beyond doubt. TCM, such as SQP containing quercetin, can also play a role in regulating PTGS2.

### 3.5. Results of Animal Experiments

#### 3.5.1. Results of Biochemical Indicators

**Figure 9:**
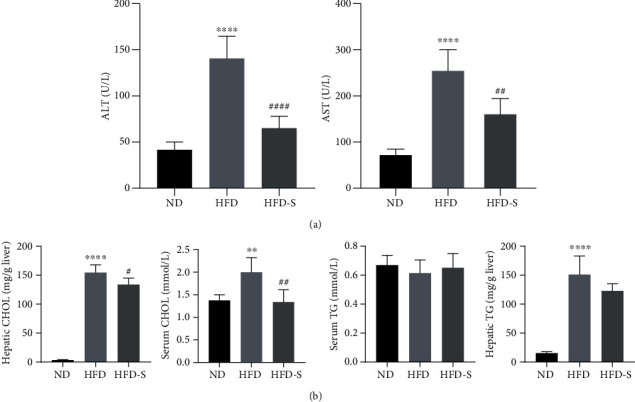
Biochemical indicator results (*n* = 5, mean ± SD). (a) The levels of ALT and AST in serum. (b) The levels of CHOL and TG in the serum and liver. ^∗∗^*p* < 0.01, ^∗∗∗∗^*p* < 0.0001 vs. ND; ^#^*p* < 0.05, ^##^*p* < 0.01, ^####^*p* < 0.0001 vs. HFD.

Compared with the rats in the ND group, the biochemical indicators of liver injury (ALT, AST) in the HFD-induced NASH rats were significantly increased (*p* < 0.05). SQP treatment significantly alleviated the liver injury in rats and restored their abnormally elevated ALT and AST levels (*p* < 0.05) (Figure 9(a)).

In addition, HFD also caused lipid metabolism disorder in rats. There was a significant increase in CHOL in the rat serum and liver after long-term HFD (*p* < 0.05), while SQP could significantly reduce CHOL (*p* < 0.05) (Figure 9(b)). The changes in serum TG in rats were not significant, which was mainly due to the characteristics of the rats. We believe that this may be due to the disorder of lipid metabolism in rats caused by HFD, causing TG to accumulate in the liver, resulting in a large amount of lipid accumulation and the formation of hepatic steatosis. The hepatic TG content results also support this conclusion. The hepatic TG content of mice in the HFD group was significantly higher than that in the ND group (*p* < 0.05). After SQP treatment, the TG content in the liver of rats was reduced. Therefore, we deem that compared with rats and mice, golden hamsters and guinea pigs with lipid profiles closer to *Homo sapiens* are more suitable for observing the metabolic changes in serum TG [26].

#### 3.5.2. Results of Hepatic Pathology

**Figure 10:**
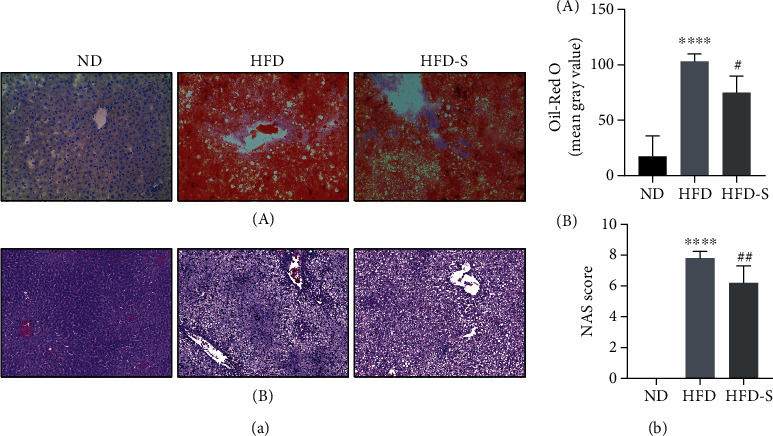
Hepatic pathology results (*n* = 5, mean ± SD). (a) The hepatic pathology results. (A) The Oil Red O staining (×100). B The HE staining (×200). (b), (A) The results of semiquantitative Oil Red O staining. (B) The NAS score results.^∗∗∗∗^*p* < 0.0001 vs. ND; ^##^*p* < 0.01 vs. HFD.

Figure 10(a)-A shows the results of the Oil Red O staining of rat livers. Fat droplets in the liver were stained red to reflect the degree of steatosis. Fat droplets accumulated significantly in liver tissues of HFD-fed NASH rats compared to normal rats. SQP intervention can dramatically reduce lipid deposition in the liver but cannot restore it to normal.

Figure 10(b)-A shows the HE staining, reflecting the hepatic inflammation of rats. The infiltration of inflammatory cells in the livers of HFD rats was significantly higher than that of normal rats. SQP reduced the infiltration of inflammatory cells and reduced the level of liver inflammation. The NAS score is based on the pathological features of NAFLD (steatosis, inflammation, ballooning) and is an index used to measure the severity of NAFLD. NAS ≥ 5 is a surrogate for the histologic diagnosis of NASH [21]. From Figure 10(b), we know that the NAS score of HFD-induced rats was significantly higher than that of normal rats (*p* < 0.05). In addition, the NAS of rats in the HFD group was greater than 5, which suggested that these rats could be diagnosed with NASH. Treatment with SQP significantly reduced the NAS score of rats (*p* < 0.05) and improved NASH.

As the gold standard for the clinical diagnosis of NASH, the pathological results combined with the previous biochemical indicators are sufficient to demonstrate the success of the animal model in this study, and that SQP can alleviate the hepatic fat accumulation and inflammation and treat NASH.

#### 3.5.3. Results of Western Blotting

**Figure 11:**
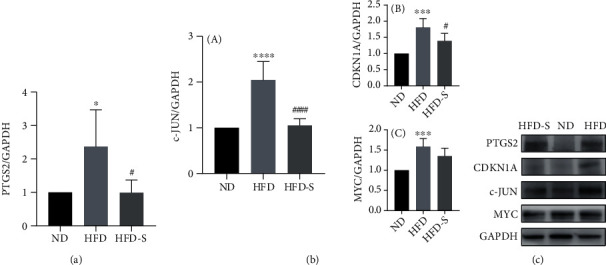
Western blotting results of hub target genes (*n* = 5, mean ± SD). (a) The protein expression of the hub target gene PTGS2. (b) The expression of the hub target gene c-JUN and its interacting proteins. (A) Protein expression of the hub target gene c-JUN. (B) Protein expression of CDKN1A. (C) The protein expression of MYC. (c) The protein expression quantitation. ^∗^*p* < 0.05, ^∗∗∗^*p* < 0.001, and ^∗∗∗∗^*p* < 0.0001 vs. ND; ^#^*p* < 0.05, ^####^*p* < 0.0001 vs. HFD.

The protein expression results of the hub target genes of SQP in treating NASH rats (PTGS2, JUN, CDKN1A, and MYC) are shown in Figure 11. Compared with the rats in the ND group, the expression of PTGS2, c-JUN, CDKN1A, and MYC in the HFD group was significantly elevated (*p* < 0.05). The expression of PTGS2, c-JUN, and CDKN1A was reduced significantly in the HFD-S group treated with SQP (*p* < 0.05). The expression of MYC decreased after SQP treatment, but the difference was not significant (*p* > 0.05).

In summary, the protein expression of the SQP hub target genes was elevated after long-term HFD feeding and downregulated by SQP treatment. SQP could regulate the protein expression of the hub genes, which further supports the reliability of the screening.

#### 3.5.4. Results of Quantitative Real-Time PCR

**Figure 12:**
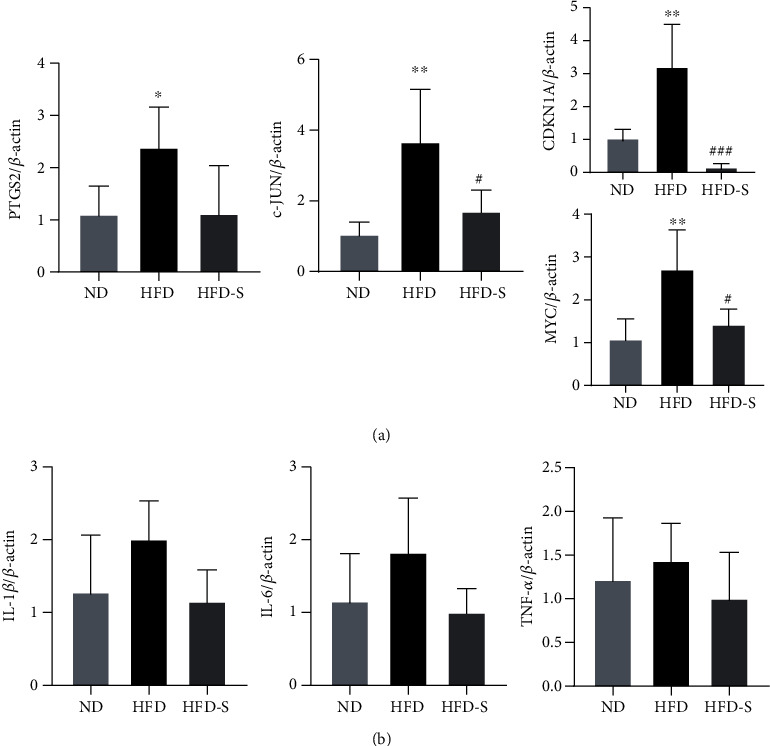
qRT–PCR results (*n* = 5, mean ± SD). (a) The RNA level of hub target genes. (b) The RNA levels of inflammatory cytokines. ^∗^*p* < 0.05, ^∗∗^*p* < 0.01 vs. ND; ^#^*p* < 0.05, ^###^*p* < 0.001 vs. HFD.

To increase the reliability of the previous analysis, we detected RNA changes in the SQP hub target genes and inflammatory cytokines by qRT–PCR.  Figure 12(a) shows that the RNA levels of MYC, CDKN1A, and c-JUN were upregulated in the HFD group and downregulated after the SQP intervention (*p* < 0.05). The RNA level of PTGS2 in the HFD group was significantly upregulated compared with that in the normal group (*p* < 0.05). After SQP intervention, the expression level showed a downwards trend but without significance (*p* > 0.05). The RNA expression levels of the SQP hub target genes were both elevated after long-term HFD feeding and downregulated with treatment, consistent with the trend of the protein levels.

We also measured the RNA levels of inflammatory cytokines (IL-1*β*, IL-6, TNF-*α*) to estimate hepatic inflammation.  Figure 12(b) shows that the RNA levels of IL-1*β*, IL-6, and TNF-*α* were upregulated in the HFD group and downregulated in the HFD-S group, but the difference was not significant (*p* > 0.05). These results showed that long-term HFD feeding promoted the secretion of inflammatory cytokines by the hepatocytes of rats, which led to hepatic inflammation. SQP could improve inflammation and treat NASH by reducing the secretion of inflammatory cytokines.

## 4. Discussion

With the improvement of the economy and the subsequent increase in the obesity rate, NAFLD, characterized by excessive accumulation of lipids in the liver, has surpassed viral hepatitis as the leading cause of chronic liver disease in China [27]. NAFLD also directly influences a quarter of adults worldwide, and there is a trend of younger episodes [28]. A critical stage in NAFLD progression, NASH may deteriorate to fibrosis and cirrhosis because of the continuous hepatocyte injury caused by inflammation [29]. Undoubtedly, treating hepatic inflammation is a valuable method to treat NASH. The research of Lan et al. further confirmed the feasibility of drugs targeting inflammation in treating NASH [30].

In this study, we confirmed that SQP could ameliorate NASH in rats by improving hepatic inflammation and simultaneously screened four hub inflammation-related target genes (PTGS2, JUN, MYC, and CDKN1A) via network pharmacology and bioinformatics analysis.

PTGS2, also known as cyclooxygenase 2 (COX-2), is an essential gene in cell death signaling. Its overexpression significantly promotes hepatocyte death, causing hepatic inflammation [31]. PTGS2 is the main enzyme causing inflammation [32]. The critical active mechanism is associated with prostaglandins (product of PTGS2), whose functions have been confirmed in diverse physiopathological processes, including activating the inflammatory response [33]. Many nonsteroidal anti-inflammatory drugs (NSAIDs), such as acetylsalicylic acid, ibuprofen, and sulindac, act as nonspecific PTGS2 inhibitors and prevent PTGS2 from converting arachidonic acid into prostaglandins to achieve anti-inflammatory effects [34].

While the tissue is under stress, its cells will produce inflammatory memory: fixing inflammation-related proteins on its deoxyribonucleic acid (DNA), remembering the workplace, and thus constructing memory regions, contributing to a rapid response of subsequent inflammation [35]. These memory regions of the genome always become accessible while suffering stimuli and remain accessible after inflammation with the regulation of transcription factors, such as signal transducer and activator of transcription 3 (STAT3), JUN, and Fos protooncogene (FOS) [36]. When the tissue suffers stress, STAT3 responds first, coordinating the inflammatory response. Then, STAT3 will give way to the roles of JUN and FOS. FOS will decline as the stimulus subsides. However, JUN continues to maintain the opened inflammatory memory regions, and it has the ability to recruit FOS to the memory region and achieve an inflammatory response when the stimulus strikes again [37].

The system can be activated unilaterally to respond to almost any stimulus but may not always benefit the affected tissues, such as the liver. Continuous guarding of the inflammatory memory regions by JUN will make the tissue try to clear even the slightest abnormality, which can easily cause continuous chronic inflammation. Persistent chronic hepatic inflammation is crucial to the occurrence of NASH, and it is the primary mechanism leading to the progression of NASH to fibrosis and cirrhosis [38].

CDKN1A and MYC were selected as hub target genes due to their direct interactions with JUN. CDKN1A is a crucial gene in ageing, also known as a biomarker of cellular ageing, and it can promote the production of senescence-associated secretory phenotype (SASP) by reducing the phosphorylation of Rb [39]. With the assistance of SASP, the immune system will more easily recognize and eliminate senescent cells. However, an excessive SASP will produce excessive inflammatory cytokines or overactivated inflammatory pathways, causing abnormal inflammation [40].

Kalra and Kumar found that MYC is an essential gene in regulating the apoptosis of cells [41]. The apoptotic body produced by apoptosis is the target of phagocytosis and clearance by phagocytes and is a critical mechanism for causing inflammation. The overexpression of MYC causes abnormal inflammation in tissues by promoting cell apoptosis. Luo et al. suggested that the targeted inhibition of MYC is a feasible mechanism for treating NASH [42]. In addition, MYC is one of the most common protooncogenes and is upregulated in 50-60% of malignant tumors [43]. The high expression of MYC is always associated with a poor prognosis and low survival rate of cancer patients. Many studies have confirmed that the overexpression of MYC promotes tumorigenesis; in contrast, inhibiting or knocking down MYC reduces tumorigenesis [44, 45]. The MYC expression in HFD-fed rats was significantly higher than that in the control group, suggesting that tumorigenesis may be initiated, and that MYC acts as a “starting gun” gene. The use of SQP can reduce the expression of MYC. This result may suggest that SQP can help delay the progression to HCC or even be directly used to treat HCC.

In this study, we clarified the crucial mechanism of SQP in treating NASH, identified four hub target genes, and completed the verification. These results may provide theoretical support for promoting the clinical usage of SQP in NASH treatment. We have discovered the potential feasibility of treating HCC with SQP by inhibiting the expression of MYC, but this has not been verified. SQP may treat both NASH and HCC by targeting a common mechanism.

This result is consistent with the advantages of TCM formulas that treat diseases via multiple targets and channels. Because they contain many Chinese herbs and various active compounds, it is not easy to fully clarify the therapeutic principles of TCM formulas. However, this is precisely the advantage of TCM formulas: TCM formulas are not limited to treating single or similar diseases. TCM has a history of thousands of years, and formulas, the essence of TCM, are undoubtedly a treasure trove of undiscovered drugs. We should strengthen their research and applications.

## 5. Conclusion

(1) Our study verified that it is feasible and reliable to screen the therapeutic targets of TCM through network pharmacology analysis. (2) Combining the screening of network pharmacology and the validation of animal experiments, we confirmed that SQP could improve excess lipid accumulation and inflammation in the rat liver and treat NASH rats. (3) Quercetin may be an essential active compound for SQP to exert its curative effect. (4) PTGS2, JUN, MYC, and CDKN1A are the hub target genes of SQP in the treatment of NASH rats.

## Figures and Tables

**Table 1:** mRNA primer sequences.

Gene	Gene ID	Primers (5′⟶3′)	
*β*-Actin	81822	Sense	TGCTGTCACCTTCACCGTTC
		Antisense	GTCCACCGCAAATGCTTCTA
PTGS2	29527	Sense	TGAACACGGACTTGCTCACTTTG
		Antisense	AGGCCTTTGCCACTGCTTGTA
MYC	24577	Sense	ATGCCCCTCAACGTGAACTTC
		Antisense	GTCGCAGATGAAATAGGGCTG
CDKN1A	114851	Sense	GAGCAAAGTATGCCGTCGTC
		Antisense	CTCAGTGGCGAAGTCAAAGTTC
JUN	24516	Sense	ATCGCTGCCTCCAAGTGC
		Antisense	CTGTGCCACCTGTTCCCTG
TNF-*α*	24835	Sense	GCGTGTTCATCCGTTCTCTAC
		Antisense	GTCTCGTGTGTTTCTGAGCAT
IL-6	24498	Sense	ATTGTATGAACAGCGATGATGCA
		Antisense	CCAGGTAGAAACGCAACTCCAGA
IL-1*β*	24494	Sense	AGGAGAGACAAGCAACGACAA
		Antisense	GTTTGGGATCCACACTCTCCA

**Table 2:** Top 5 results of enrichment analysis of BP, CC, MF, and the pathways of SQP.

Ontology	ID	Description	GeneRatio	BgRatio	*p* value	*p*.adjust	*q* value
BP	GO: 0032496	Response to lipopolysaccharide	6/8	330/18670	7.92*e*-10	6.31*e*-07	1.47*e*-07
BP	GO: 0002237	Response to molecule of bacterial origin	6/8	343/18670	9.99*e*-10	6.31*e*-07	1.47*e*-07
BP	GO: 0150076	Neuroinflammatory response	4/8	75/18670	1.66*e*-08	6.99*e*-06	1.63*e*-06
BP	GO: 0010575	Positive regulation of vascular endothelial growth factor production	3/8	29/18670	1.88*e*-07	5.54*e*-05	1.29*e*-05
BP	GO: 0002675	Positive regulation of acute inflammatory response	3/8	31/18670	2.31*e*-07	5.54*e*-05	1.29*e*-05
MF	GO: 0005125	Cytokine activity	3/8	220/17697	1.01*e*-04	0.006	0.002
MF	GO: 0005126	Cytokine receptor binding	3/8	286/17697	2.20*e*-04	0.006	0.003
MF	GO: 0033613	Activating transcription factor binding	2/8	85/17697	6.27*e*-04	0.012	0.005
MF	GO: 0048018	Receptor ligand activity	3/8	482/17697	0.001	0.015	0.006
MF	GO: 0070851	Growth factor receptor binding	2/8	134/17697	0.002	0.018	0.007
KEGG	hsa04657	IL-17 signaling pathway	5/8	94/8076	1.05*e*-08	4.75*e*-07	1.87*e*-07
KEGG	hsa05163	Human cytomegalovirus infection	6/8	225/8076	1.17*e*-08	4.75*e*-07	1.87*e*-07
KEGG	hsa04933	AGE-RAGE signaling pathway in diabetic complications	5/8	100/8076	1.43*e*-08	4.75*e*-07	1.87*e*-07
KEGG	hsa05142	Chagas disease	5/8	102/8076	1.58*e*-08	4.75*e*-07	1.87*e*-07
KEGG	hsa04668	TNF signaling pathway	5/8	112/8076	2.54*e*-08	6.10*e*-07	2.41*e*-07

**Table 3:** The results of molecular docking.

Compound (ligand)	Protein (receptor)	Minimum binding energy/kcal·Mol^−1^
Quercetin (MOL000098)	PTGS2 (1CVU)	-9.1
	JUN (1JNM)	-5.3
	MYC (6G6J)	-6.5
	CDKN1A (4RJF)	-4.7

## Data Availability

The original data for the study can be acquired from the corresponding authors upon request.

## Abbreviations

ALT: Alanine aminotransferase
AST: Aspartate aminotransferase
BP: Biological process
CC: Cellular component
CCL2: C-C motif chemokine ligand 2
CDKN1A: Cyclin-dependent kinase inhibitor 1A
CHOL: Total cholesterol
DEGs: Differentially expressed genes
DL: Drug-like
DM: Diabetic mellitus
DNA: Deoxyribonucleic acid
FOS: Fos protooncogene
GO: Gene Ontology
HCC: Hepatocellular carcinoma
HFD: High-fat diet
HFD-S: High-fat diet with SQP
HE: Hematoxylin-eosin staining
HDL-c: High-density lipoprotein cholesterol
IL1B: Interleukin 1 beta
IL6: Interleukin 6
LDL-c: Low-density lipoprotein cholesterol
MYC: MYC protooncogene
JUN: Jun oncogene
KEGG: Kyoto Encyclopedia of Genes and Genomes
NAFL: Nonalcoholic fatty liver
NAFLD: Nonalcoholic fatty liver disease
NAS: NAFLD Activity Score
NASH: Nonalcoholic steatohepatitis
ND: Normal diet
NSAIDs: Nonsteroidal anti-inflammatory drugs
MF: Molecular function
OB: Oral bioavailability
PDB: Protein Data Bank
PPI: Protein-protein interaction
PTGS2: Prostaglandin-endoperoxide synthase 2
qRT-PCR: Quantitative real-time PCR
SASP: Senescence-associated secretory phenotype
SERPINE1: Serpin family E member 1
SQP: Shenqi pill
STAT3: Signal transducer and activator of transcription 3
TCM: Traditional Chinese medicine
TCMSP: Traditional Chinese Medicine Database and Analysis Platform
TG: Triglyceride
UPLC-MS/MS: Ultraperformance liquid chromatography-quadrupole/time-of-flight mass spectrometry.

## Supplementary Materials

Supplementary Materials Supplementary Table 1: the information of selected SQP active compounds.
