# Supplementary material for: Exploring and Verifying the Mechanism and Targets of Shenqi Pill in the Treatment of Nonalcoholic Steatohepatitis via Network Pharmacology and Experiments
Source: J Immunol Res. 2022 Jun 12;2022:6588144. doi: 10.1155/2022/6588144 (PMC9207021; doi:10.1155/2022/6588144)
Supplement: Supplementary Materials — Supplementary Table 1: the information of selected SQP active compounds. [file 6588144.f1.docx]

**Supplementary Table 1:** The information of selected SQP active ingredients.

| **Mol ID** | **Molecule name** | **Molecular weight** | | **OB(%)** | **DL** |
| --- | --- | --- | --- | --- | --- |
| **1.Fulin/Poria Cocos(Schw.) Wolf.** | | | | | |
| MOL000282 | ergosta-7,22E-dien-3beta-ol | | 398.74 | 43.51 | 0.72 |
| MOL000291 | Poricoic acid B | | 484.74 | 30.52 | 0.75 |
| MOL000292 | poricoic acid C | | 482.77 | 38.15 | 0.75 |
| MOL000296 | hederagenin | | 414.79 | 36.91 | 0.75 |
| MOL000290 | Poricoic acid A | | 498.77 | 30.61 | 0.76 |
| MOL000279 | Cerevisterol | | 430.74 | 37.96 | 0.77 |
| MOL000275 | trametenolic acid | | 456.78 | 38.71 | 0.8 |
| MOL000287 | 3beta-Hydroxy-24-methylene-8-lanostene-21-oic acid | | 470.81 | 38.7 | 0.81 |
| MOL000289 | pachymic acid | | 528.85 | 33.63 | 0.81 |
| MOL000276 | 7,9(11)-dehydropachymic acid | | 526.83 | 35.11 | 0.81 |
| MOL000283 | Ergosterol peroxide | | 430.74 | 40.36 | 0.81 |
| MOL000273 | (2R)-2-[(3S,5R,10S,13R,14R,16R,17R)-3,16-dihydroxy-4,4,10,13,14-pentamethyl-2,3,5,6,12,15,16,17-octahydro-1H-cyclopenta[a]phenanthren-17-yl]-6-methylhept-5-enoic acid | | 470.76 | 30.93 | 0.81 |
| MOL000280 | (2R)-2-[(3S,5R,10S,13R,14R,16R,17R)-3,16-dihydroxy-4,4,10,13,14-pentamethyl-2,3,5,6,12,15,16,17-octahydro-1H-cyclopenta[a]phenanthren-17-yl]-5-isopropyl-hex-5-enoic acid | | 484.79 | 31.07 | 0.82 |
| MOL000285 | (2R)-2-[(5R,10S,13R,14R,16R,17R)-16-hydroxy-3-keto-4,4,10,13,14-pentamethyl-1,2,5,6,12,15,16,17-octahydrocyclopenta[a]phenanthren-17-yl]-5-isopropyl-hex-5-enoic acid | | 482.77 | 38.26 | 0.82 |
| MOL000300 | dehydroeburicoic acid | | 453.75 | 44.17 | 0.83 |
| **2.Fuzi/Aconiti Lateralis Radix Praeparata** | | | | | |
| MOL002211 | 11,14-eicosadienoic acid | | 308.56 | 39.99 | 0.2 |
| MOL002388 | Delphin_qt | | 303.26 | 57.76 | 0.28 |
| MOL002392 | Deltoin | | 328.39 | 46.69 | 0.37 |
| MOL002393 | Demethyldelavaine A | | 700.91 | 34.52 | 0.18 |
| MOL002394 | Demethyldelavaine B | | 700.91 | 34.52 | 0.18 |
| MOL002395 | Deoxyandrographolide | | 334.5 | 56.3 | 0.31 |
| MOL002397 | karakoline | | 377.58 | 51.73 | 0.73 |
| MOL002398 | Karanjin | | 292.3 | 69.56 | 0.34 |
| MOL002401 | Neokadsuranic acid B | | 452.74 | 43.1 | 0.85 |
| MOL002406 | 2,7-Dideacetyl-2,7-dibenzoyl-taxayunnanine F | | 776.9 | 39.43 | 0.38 |
| MOL002410 | benzoylnapelline | | 463.67 | 34.06 | 0.53 |
| MOL002415 | 6-Demethyldesoline | | 453.64 | 51.87 | 0.66 |
| MOL002416 | deoxyaconitine | | 629.82 | 30.96 | 0.24 |
| MOL002419 | (R)-Norcoclaurine | | 271.34 | 82.54 | 0.21 |
| MOL002421 | ignavine | | 449.59 | 84.08 | 0.25 |
| MOL002422 | isotalatizidine | | 407.61 | 50.82 | 0.73 |
| MOL002423 | jesaconitine | | 675.85 | 33.41 | 0.19 |
| MOL002433 | (3R,8S,9R,10R,13R,14S,17R)-3-hydroxy-4,4,9,13,14-pentamethyl-17-[(E,2R)-6-methyl-7-[(2R,3R,4S,5S,6R)-3,4,5-trihydroxy-6-[[(2R,3R,4S,5S,6R)-3,4,5-trihydroxy-6-(hydroxymethyl)oxan-2-yl]oxymethyl]oxan-2-yl]oxyhept-5-en-2-yl]-1,2,3,7,8,10,12,15,16,17-decahydr | | 781.1 | 41.52 | 0.22 |
| MOL002434 | Carnosifloside I_qt | | 456.78 | 38.16 | 0.8 |
| MOL000359 | sitosterol | | 414.79 | 36.91 | 0.75 |
| MOL000538 | hypaconitine | | 615.79 | 31.39 | 0.26 |
| **3.Guizhi/Cinnamomi Ramulus** | | | | | |
| MOL001736 | (-)-taxifolin | | 304.27 | 60.51 | 0.27 |
| MOL000358 | beta-sitosterol | | 414.79 | 36.91 | 0.75 |
| MOL000359 | sitosterol | | 414.79 | 36.91 | 0.75 |
| MOL000492 | (+)-catechin | | 290.29 | 54.83 | 0.24 |
| MOL000073 | ent-Epicatechin | | 290.29 | 48.96 | 0.24 |
| MOL004576 | taxifolin | | 304.27 | 57.84 | 0.27 |
| MOL011169 | Peroxyergosterol | | 428.72 | 44.39 | 0.82 |
| **4.Mudanpi/Cortex Moutan** | | | | | |
| MOL000422 | kaempferol | | 286.25 | 41.88 | 0.24 |
| MOL000492 | (+)-catechin | | 290.29 | 54.83 | 0.24 |
| MOL000098 | quercetin | | 302.25 | 46.43 | 0.28 |
| MOL007374 | 5-[[5-(4-methoxyphenyl)-2-furyl]methylene]barbituric acid | | 312.3 | 43.44 | 0.3 |
| MOL007384 | paeonidanin_qt | | 330.41 | 65.31 | 0.35 |
| MOL007382 | mudanpioside-h_qt 2 | | 336.37 | 42.36 | 0.37 |
| MOL001925 | paeoniflorin_qt | | 318.35 | 68.18 | 0.4 |
| MOL007369 | 4-O-methylpaeoniflorin_qt | | 332.38 | 67.24 | 0.43 |
| MOL007003 | benzoyl paeoniflorin | | 584.62 | 31.14 | 0.54 |
| MOL000359 | sitosterol | | 414.79 | 36.91 | 0.75 |
| MOL000211 | Mairin | | 456.78 | 55.38 | 0.78 |
| **5.Shanyao/Rhizoma Dioscoreae** | | | | | |
| MOL001559 | piperlonguminine | | 273.36 | 30.71 | 0.18 |
| MOL001736 | (-)-taxifolin | | 304.27 | 60.51 | 0.27 |
| MOL000310 | Denudatin B | | 356.45 | 61.47 | 0.38 |
| MOL000322 | Kadsurenone | | 356.45 | 54.72 | 0.38 |
| MOL005429 | hancinol | | 372.5 | 64.01 | 0.37 |
| MOL005430 | hancinone C | | 400.51 | 59.05 | 0.39 |
| MOL005435 | 24-Methylcholest-5-enyl-3belta-O-glucopyranoside_qt | | 400.76 | 37.58 | 0.72 |
| MOL005438 | campesterol | | 400.76 | 37.58 | 0.71 |
| MOL005440 | Isofucosterol | | 412.77 | 43.78 | 0.76 |
| MOL000449 | Stigmasterol | | 412.77 | 43.83 | 0.76 |
| MOL005458 | Dioscoreside C_qt | | 444.72 | 36.38 | 0.87 |
| MOL000546 | diosgenin | | 414.69 | 80.88 | 0.81 |
| MOL005461 | Doradexanthin | | 584.96 | 38.16 | 0.54 |
| MOL005463 | Methylcimicifugoside_qt | | 556.81 | 31.69 | 0.24 |
| MOL005465 | AIDS180907 | | 394.45 | 45.33 | 0.77 |
| MOL000953 | CLR | | 386.73 | 37.87 | 0.68 |
| **6.Shanzhuyu/Cornus Officinalis Sieb. Et Zucc.** | | | | | |
| MOL001494 | Mandenol | | 308.56 | 42 | 0.19 |
| MOL001495 | Ethyl linolenate | | 306.54 | 46.1 | 0.2 |
| MOL001771 | poriferast-5-en-3beta-ol | | 414.79 | 36.91 | 0.75 |
| MOL002879 | Diop | | 390.62 | 43.59 | 0.39 |
| MOL002883 | Ethyl oleate (NF) | | 310.58 | 32.4 | 0.19 |
| MOL003137 | Leucanthoside | | 462.44 | 32.12 | 0.78 |
| MOL000358 | beta-sitosterol | | 414.79 | 36.91 | 0.75 |
| MOL000359 | sitosterol | | 414.79 | 36.91 | 0.75 |
| MOL000449 | Stigmasterol | | 412.77 | 43.83 | 0.76 |
| MOL005360 | malkangunin | | 432.56 | 57.71 | 0.63 |
| MOL005481 | 2,6,10,14,18-pentamethylicosa-2,6,10,14,18-pentaene | | 342.67 | 33.4 | 0.24 |
